# Self-reported Use and Attitudes Regarding Herbal Medicine Safety During Pregnancy in Iran

**Published:** 2012-05-28

**Authors:** Mohammadreza Sattari, Maryam Dilmaghanizadeh, Hadi Hamishehkar, Simin Ozar Mashayekhi

**Affiliations:** 1 Infectious Disease Research Center, Tabriz University of Medical Sciences, IR Iran; 2 Student Research Center, Faculty of Pharmacy, Tabriz University of Medical Sciences, IR Iran; 3 Drug applied research center, Faculty of Pharmacy, Tabriz University of Medical Sciences, IR Iran; 4 Health Services Management Research Center, Faculty of Pharmacy, Tabriz University of Medical Sciences, IR Iran

**Keywords:** Pregnancy, Fetus, Safety, Plants, Medicinal

## Abstract

**Background:**

Despite the vast use of herbal medicines in the world, little is known about their use in pregnancy and the attitudes of pregnant women regarding their safety during pregnancy.

**Objectives:**

The aim of this study was to evaluate the use and attitudes of pregnant women toward herbal medicine use in pregnancy in Iran.

**Materials and Methods:**

A questionnaire was completed by 400 women selected by convenience sampling from postnatal and prenatal wards of two hospitals. Data were analyzed using the SPSS software version 13.5. Chi2 test was used to analyze the data.

**Results:**

The median age was 26.4 (± 5.2) years and the mean number of pregnancies was 1.9 (± 0.98). The use of herbal remedies during pregnancy was positive in 22.3% of patients. They took herbal medicines recommended by their physician (46.1%), through self-medication (44.9%), or with the advice of family members or friends (9%). Additionally, 39.8% believed that it was safe to use herbal remedies during pregnancy, 32.3% believed that it was harmful for both mother and fetus, and 22% did not know whether it was safe or not.

**Conclusions:**

Herbal medicine use was not high among our subjects but was significantly affected by age. The level of education, place of living, and number of pregnancies significantly affected the attitudes of the subjects. Women with higher education mostly relied on their own information, whereas those with lower education relied on physician advice. Further educational programs are required to increase the information for this group of susceptible individuals.

## 1. Background

Herbal remedies are used throughout the world (1) and women frequently consume herbal medicines during their pregnancy (2). In the past, herbs were the original sources of most drugs. Today we are witnessing an increase in the use of herbal remedies throughout the world (3), which raises the question of how safe these preparations are for an unborn fetus. Pregnant women may use particular herbs to treat nausea and vomiting (4) and gastric reflux (5).

## 2. Objectives

In the present study, our aim was to examine the level of awareness of pregnant women regarding the safety of herbal medicines during pregnancy in Tabriz city, which is located in the northwest of Iran. By determining the level of awareness among this group, we could plan further educational programs.

## 3. Patients and Methods

The data were collected from 400 women undergoing postnatal and prenatal care in the maternity units of two hospitals—one private and one public. The sampling was performed by a convenience sampling method. The subjects were provided with written information and those who signed a consent form enrolled in the study.

The questionnaire was prepared in Persian; however, one of the investigators was available to help for subjects who could not read or speak Persian. The questionnaire was divided into 2 sections. The first section included demographic information and the second section asked questions about herbal medicine use, the recommender of herbal medicines during pregnancy, and the attitudes of subjects on the safety of herbal medicines in pregnancy. The procedures were in accordance with the ethical standards of our university and regional committee on human experimentation. The patients’ confidentiality, privacy, and rights were protected. SPSS software (version 14) and Chi^2^ test were used to analyze the data. All tests were conducted at the *P* < 0.05 level of significance.

## 4. Results

**Figure 1:**
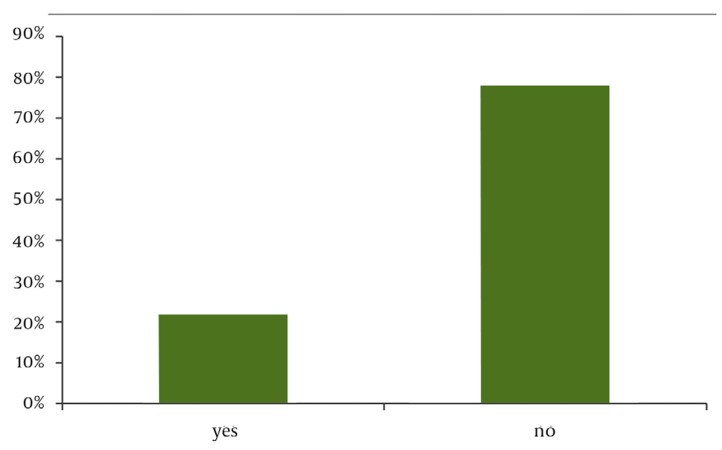
The Use of Herbal Remedies During Pregnancy.

**Figure 2:**
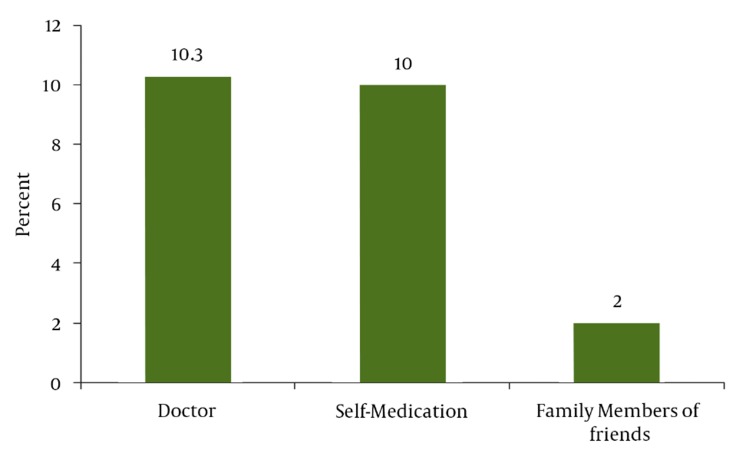
The Recommender of Herbal Medicines During Pregnancy

**Figure 3:**
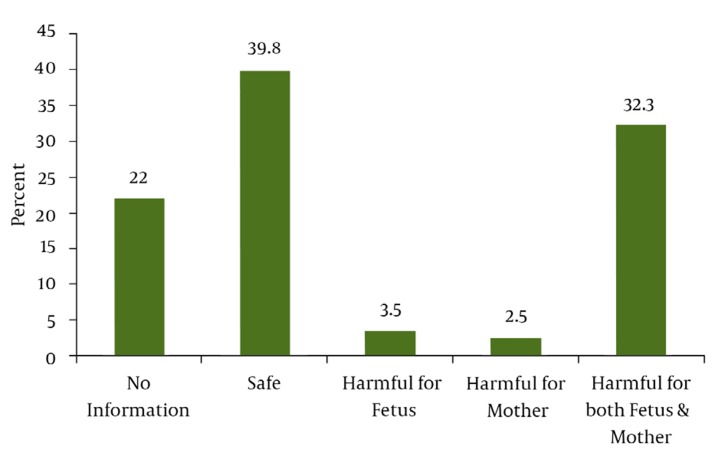
Attitude of the Subjects on Safety of Herbal Medicines Use During Pregnancy.

Sampling was performed between August 2006 and May 2007. Four hundred subjects with a median age of 26.4 (± 5.2) years, with 183 (45.8%) in their first pregnancy, 118 (29.5%) in their second pregnancy, and 99 (24.7%) with two or more previous pregnancies, entered the study. Fifty-three percent had a university education, 72% lived in cities, and 84% had no history of illness. The use of herbal remedies during pregnancy was positive in 89 (22.3%) subjects (Figure 1). The recommenders of herbal medicine use were the subjects’ physicians (46.1%) or family members/friends (9%). Additionally, 44.9% reported self-medicated herbal medicine use. Pharmacists played no role in the consumption of herbal medicines (Figure 2). Among 400 participants, 159 (39.8%) believed that it was safe to use herbal remedies during pregnancy, 129 (32.3%) believed it was harmful for both mother and fetus, and 88 (22%) did not know whether it was safe or not. Further, 3.5% and 2.5% believed that it was harmful for the fetus and mother, respectively (Figure 3) (Table 1).

**Table 1:** Demographic Information of the Study Population.

Demographic Characteristics of the subjects	No,%
Women Age, years	
15-19	20, 5.0
20-24	127, 31.8
25-29	160, 40.0
30-34	61, 15.3
35-39	30, 7.5
40-44	2, 0.5
Number of previous pregnancy	
None	183, 45.8
One	118, 29.5
Two	69, 17.3
More	30, 7.5
Unsuccessful previous pregnancies	
Yes	17, 7.5
No	383, 92.5
Number of previous children	
None	200, 50.0
One	127, 31.8
Two	54, 13.5
More	19, 4.8
Level of Education	
High school or lower	184, 46.0
Diploma	147, 36.8
University education	69, 17.3
Area of residence	
City	289, 72.3
Village	111, 27.8
Husbands Age (Year)	
20-24	30, 7.5
25-29	115, 28.8
30-34	161, 40.3
35-39	60, 15.0
40-44	34, 8.5
History of illnesses in the mother; physical or mental	
No	337, 84.3
Yes	63, 15.8
History of illnesses in the previous child(ren)	
Yes	14, 8.2
No	157, 91.8
History of illnesses in the newborn	
No	165, 82.5
Yes	9, 4.5
Stillborn	8, 4
Pre-term	18, 9

## 5. Discussion

Herbal medicines have been used since ancient times for the treatment of a range of diseases and have stimulatory effects on innate immunity. The herbal medicine use among our subjects was similar to that of a general population in Australia (2), less than that of pregnant populations in Norway (6-8) and Australia (2), but more than pregnant populations in the USA (9).

One of the most important findings of this study was that although herbal remedies are considered safe by general population (10), only 24.5% pregnant women in our study believed in the safety of herbal remedies for the mother and/or fetus during pregnancy. Although many pregnant women consider herbal medicine to be safer than pharmacological medicines (11), it is known that the active ingredients of herbs have activity similar to that of purified medications and have the same potential to cause serious adverse effects (12). Therefore, when using herbal remedies during pregnancy, these risks should be considered. Cuzzolin and colleagues showed that users of herbal medicines were more often affected by pregnancy-related morbidity and their neonates were more frequently small for their gestational age. A higher incidence of threatening miscarriages and preterm labor was observed among regular users of chamomile and licorice (13). Although the information on safety of herbal medicines during pregnancy is not complete, it is safe to advise pregnant mothers not to expose their unborn child to the risk of herbal medicines. The main recommender of herbal medicines was the physician, whereas self-medication was the most frequent in Australia (2) and word-of-mouth communication in Canada (11).

We found a significant relationship between age and use of herbal medicines, where subjects aged between 20 and 29 years reported the highest use of herbal medicines. The number of pregnancy and children also had a significant relationship with herbal medicine use, as women in their first pregnancy were mostly nonusers in this study. Westfall found no relationship between age or gravidity and the knowledge of pregnant women regarding herbal medicines. Rahman and coworkers found no relationship between socioeconomic factors and use of herbal medicines, (14) whereas the prior use of herbs, knowledge about herbal medicines and age between 26 and 35 years were factors associated with the use of herbal medicines during pregnancy (8). In the present study, no significant relationship was observed between the place of living and the use of herbal remedies, but there was a relationship between the place of living and the positive attitudes of the subjects towards the effects of herbal remedies on the mother and fetus. The place of living was also related to the recommender of herbal medicine use. Subjects living in cities had positive attitudes towards herbal medicines and mainly self-medicated, but subjects living in villages reported their physicians as the recommender. This finding was unexpected but a possible result of the family physician system, which is prevalent in Iranian villages but not in cities, and relies on the trust between village women and their physicians. Women with lower education were more likely to seek advice from their physicians.

Furthermore, our findings showed that there was no significant relationship between the attitudes and the use of herbal remedies during pregnancy, which was in contrast to the findings of Rahman and coworkers, who showed that users of herbal medicines had more positive attitudes towards the use of herbal medicines (15).

Although the public considers these products as traditional medicines or natural food supplements, they could be harmful during pregnancy. Therefore, all pregnant women should be educated about the safety of these drugs during pregnancy and should inform their herbal medicine provider of their pregnancy. The findings of this study may be used for health promotional interventions. Future studies should include such issues in the design of relevant research. This study evaluated the general attitudes of the subjects towards herbal medicines but did not investigate the knowledge of the subjects or if the herbal medicine use was related to the pregnancy itself. Future studies could aim to examine the knowledge of pregnant women regarding the safety of the use of specific herbs and pregnancy-related herbal medicine use.
